# Comparison of adaptive neuro-fuzzy inference system and artificial neutral networks model to categorize patients in the emergency department

**DOI:** 10.1186/2193-1801-2-416

**Published:** 2013-08-29

**Authors:** Dhifaf Azeez, Mohd Alauddin Mohd Ali, Kok Beng Gan, Ismail Saiboon

**Affiliations:** Department of Electrical, Electronic and Systems Engineering, Faculty of Engineering & Built Environment, Universiti Kebangsaan Malaysia, Bangi, Malaysia; Institute of Space Science, Universiti Kebangsaan, Malaysia, Bangi, Malaysia; Department of Emergency Medicine, Jalan Yaacob Latif, Bandar Tun Razak, 56000 Cheras, Kuala Lumpur Malaysia

**Keywords:** Emergency medical services, Triage, Neural network, Adaptive neuro-fuzzy inference system

## Abstract

Unexpected disease outbreaks and disasters are becoming primary issues facing our world. The first points of contact either at the disaster scenes or emergency department exposed the frontline workers and medical physicians to the risk of infections. Therefore, there is a persuasive demand for the integration and exploitation of heterogeneous biomedical information to improve clinical practice, medical research and point of care. In this paper, a primary triage model was designed using two different methods: an adaptive neuro-fuzzy inference system (ANFIS) and artificial neural network (ANN).When the patient is presented at the triage counter, the system will capture their vital signs and chief complains beside physiology stat and general appearance of the patient. This data will be managed and analyzed in the data server and the patient’s emergency status will be reported immediately. The proposed method will help to reduce the queue time at the triage counter and the emergency physician’s burden especially duringdisease outbreak and serious disaster. The models have been built with 2223 data set extracted from the Emergency Department of the Universiti Kebangsaan Malaysia Medical Centre to predict the primary triage category. Multilayer feed forward with one hidden layer having 12 neurons has been used for the ANN architecture. Fuzzy subtractive clustering has been used to find the fuzzy rules for the ANFIS model. The results showed that the RMSE, %RME and the accuracy which evaluated by measuring specificity and sensitivity for binary classificationof the training data were 0.14, 5.7 and 99 respectively for the ANN model and 0.85, 32.00 and 96.00 respectively for the ANFIS model. As for unseen data the root mean square error, percentage the root mean square error and the accuracy for ANN is 0.18, 7.16 and 96.7 respectively, 1.30, 49.84 and 94 respectively for ANFIS model. The ANN model was performed better for both training and unseen data than ANFIS model in term of generalization. It was therefore chosen as the technique to develop the primary triage prediction model. This primary triage model will be combined with the secondary triage prediction model to produce the final triage category as a tool to assist the medical officer in the emergency department.

## Introduction

Triage is an essential function in the Emergency Department of the hospital. It should be done within a very short time approximately two to three minutes (San Pedro et al. 2004) to sort the patients into the most appropriate assessment and treatment area. It is a process to categorize the casualties, based on their need for medical attention (Wilk et al. 2005; Sadeghi et al. 2006; Michalowski et al. 2005). Triage is the first point of direct public contact and it is very susceptible to the transmission signs of infectious diseases. For example, infectious diseases, such as SARS, Avian Flu and H1N1, are becoming serious global problems compared to the past. As a result of rapid population growth and increased mobility among people, these diseases spread quickly and vigorously to a level where the public health services may not be equipped to deal with further outbreaks.

Triage decision-making is often very complex, but is an important task to be completed for each patient attended to in the emergency department. The triage officer’s judgment, experience, patients’ clinical history, and resource availability further contribute to the complexity of triage decision-making. The issue faced by the emergency department physician is to quickly and accurately identify those patients who require more attention without overburdening the surgeon with the non-emergency problems. The emergency department physician can quickly intervene in the identified emergency cases before the patients collapse.

There are many triage systems have been implemented in the emergency department and the output has three to five categories. The triage category consists of resuscitation, emergency, urgent, non-urgent and referred. In United States, the emergency department uses the Emergency Severity Index (ESI) (Gilboy et al. 2005) for triage acuity assessment The Manchester triage system (MTS) is widely used in United Kingdom accident and emergency (A&E) departments. It has five triage categories and based on the expert opinions (Kevin 1997). The Canadian system (START) has four triage categories. It is simple triage and rapid treatment system jointly developed by the Newport Beach Fire and Marine Department and Hoag Hospital. In Malaysia, the Emergency Medicine Department at Serdang Hospital uses three categories triage system (Lee 2011). In the Emergency Department, Universiti Kebangsaan Malaysia Medical Center (UKMMC), the triage process is done manually by the triage officer using the Objective Primary Triage Scale (OPTS).

OPTS is a locally-developed system by one of the authors of this paper. It is based on ESI to quickly categorize the patients presented to the emergency department counter. However, the OPTS is paper-based and still requires a clinical specialist to categorize patients into the appropriate triage category based on the developed guidelines. This manual system, though simple, does not benefit from the power of the modern computer facilities, and leads to inconsistencies and errors. Basically, triage is performed based on the chief complaints and physiological status of the patients. Chief complaints are usually a free text input in a manual system that can be identified as the reason to visit the emergency department. Therefore, many works have been done to standardize the chief complaints, for example, Data Elements for Emergency Departments (DEEDS) 1.0 (Bradley 1996,1995). Another work to standardize the chief complaints is the Canadian Emergency Department Information System (CEDIS) (Grafstein et al. 2008).

Nowadays, expert systems and soft computing are used in many applications and one of them is medical applications. Where it is a computer program is designed to model the problem-solving ability of a human expert. There are many well-known advantages to use computerized tools and expert systems, such as reduction of missing data, better collection of data, no omission of questions, no data transcription and broader coverage of diagnoses. It is envisaged that by providing decision support tools to assist the triage officer in making correct and timely triage decisions that are consistent with standard triage scales can contribute to the improvement in the quality of life for patients and also reduce costs occurring from mistreatment (San Pedro et al. 2004).

Various mobile and handheld devices have been developed to facilitate the emergency department physician, and depend on different triage standards. (Michalowski et al. 2003) developed the Mobile Emergency Triage (MET) system for pediatric emergency service using a mobile device. It uses rough set theory and fuzzy technique to extract the rules of the incomplete data set. The overall mean accuracy of the MET system was slightly lower, but not statistically different from the accuracy of the emergency department physicians (70.2% for physicians vs. 67.2% for MET) (Michalowski et al. 2005). ITriage(Padmanabhan et al. 2006) is another mobile triage system that has a mean accuracy of 67% with a standard deviation of 29%, compared to the one using physiological lists, which has a mean accuracy of 53% with a standard deviation of 23%. The disadvantages of the mobile devices are limited memory and required an internet connection to the data server.

Sadeghi et al. (Sadeghi et al. 2006) had developed a decision support system for emergency triage using a Bayesian network. This system was developed using clinical data extracted from 90 patients with non-traumatic abdominal pain. It has a higher sensitivity (90% versus 64%) and a lower level of specificity compared to a human physician (25% versus 48%). Lin et al. (Lin et al. 2010) had developed an expert system for abnormal diagnosis of emergency triage that used cluster analysis (Ward’s method and k-means) and decision tree methods. This system depended on the saved data, and was not tested to determine the accuracy of the system with the new data. This system used limited data and did include a patient description. The cluster analysis and the rough set theory were used (Lin et al. 2011) to extract rules from the data in the emergency department. The system was followed along with the patients until they were discharged from hospital. It classified the patient into five categories with an accuracy of 0.937.

The main objective of this project is to develop an intelligent triage system with minimal human expert intervention in an emergency department, UKMMC. This system consists of two models as in OPTS. They are primary and secondary to give the final triage models. The objective of this paper is to examine the feasibility of the ANFIS which is not explored previously in categorizing the primary triage patients and neural network, depending on the primary triage section of the OPTS in the Emergency Department, UKMMC and choose the best method to build the prediction model.

### Development of primary triage model

#### ***Data collection and assessment***

The type of clinical research for this study is a retrospective study. In medicine, it is a study that looks backward in time, usually using medical records for patients who are already known to have a disease. Retrospective study can help in determinations about cause and effect and the factors that influence the outcome. The data were extracted from a primary triage section of the OPTS in the Emergency Department, UKMMC. This study was granted the ethical committee approval from the research ethics committee UKMMC. The exclusion criteria in this study were patients below 12 years old, patients who do not have any vital signs at the presentation at the triage counter, in the resuscitation triage, or died at the triage counter.

In the OPTS, primary triage is a checklist that consists of general appearance and physiological data, and respiratory rate and heart rate as the objective data. Subjective data is the chief complaint with a free text input. The attributes in the primary triage that were used as the input to the system are the general appearance and physiological data listed as a questionnaire. Chief complaint with free text input is one of the variables in developing the primary triage model. In this work, the chief complaint was coded into numerical format according to the CEDIS system. CEDIS is the most standardized and comprehensive system to code the chief complaints. The general terms of the chief complaint used in the OPTS were added into CEDIS lists with acustomized numbers to satisfy the need of model development. This primary triage model was developed using 2223 samples extracted from the OPTS. These samples were formatted into 20 columns as input and one column as output for triage category. The triage categories are resuscitation, emergent and non-urgent.

#### ***Triage prediction model***

There intelligent information processing model such as artificial neural network (ANN) and adaptive neuro fuzzy inference system (ANFIS), have been successfully implemented in medicine to develop a predictive model. ANN has been used as clinical functions of diagnosis, prognosis and survival analysis in the medical domains of oncology, critical care, and cardiovascular medicine (Lisboa 2002). Another set of applications for neural networks in medicine have been proposed by Pandey and Mishra (Pandey and Mishra 2009). ANFIS has been successfully implemented in medicine domain as in reported by several authors (Guler and Ubeyli 2005; Özkan et al. 2010; Majumdar 2011).

Neural network is one of the information processing model inspired by the way biological neural systems process data. In this study, neural network was chosen to test the applicability of the machine learning technique using triage data due to its simplicity. However, there are many parameters need to be optimized in order to achieve the desired performance using neural network model. Besides that the most effective features and its relationship with the prediction model need to be investigated. This information will not be generated by the neural network model. As for comparison purpose, ANFIS was chosen as it has capability to seek for the effective features and develop the prediction model.

In this work both ANN and ANFIS were used to develop triage prediction model. The triage data were divided into two sets based on the odd and even numbers of the patient’s identification respectively. The odd number data were used to develop the model. These data were divided randomly into 70% for training, 15% for testing, and 15% for validation to avoid bias and early termination. The even number data were used as the unseen data to measure the model generalization (Yahya et al. 2010).

##### ANNmethod

The ANN is a massively parallel structure that can learn from the knowledge base (Akay 2000). It is capable of mapping a set of one or more input to a set of one or more output to a model that can be used with a new set of data. There are two categories of neural network, which are dynamic and static (Moghaddamnia et al. 2009).

**Figure 1:**
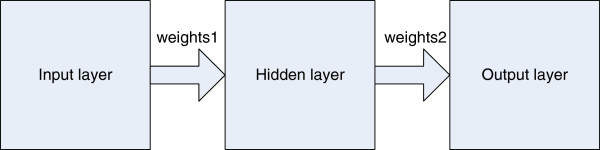
**Main blocks for ANN.**

The basic ANN consists of three layers as shown in Figure 1, where the first layer is the input layer and the input could be from one input to multiple inputs. The second layer is the hidden layer, and could be also from one to multiple hidden layers, and each layer can be set to different numbers of neurons. The last block is the output layer and the number of the output could be one to multiple outputs. The hidden layer is connected to the input and output layer through different sets of weights.

The topology could be one layer network for the linear problem and multiple layers of the nonlinear problem (Negnevitsky 2005). The most efficient and common architecture used in anANN is the feed forward ANN (Andriulli et al. 2003; Das et al. 2003; ZareNezhad and Aminian 2010). The inputs multiplied by the weights should be passed to a binary function according to the digital characteristic of the spikes, which are the elementary units of neural signal transmission. This function calls the activation function. The practical activation function used by the neuron is the step, sign, linear, and sigmoid functions. The sigmoid function is differentiable for all values of the inputs to allow the use of powerful back-propagation learning algorithms (Patterson and Draper 1998)and was chosen as an activation function for all inputs. The output activation function is set to linear function. Different training algorithms (Mangalampalli et al. 2006) and activation functions can be chosen to design different architectures and topologies of the ANN to suit the input data.

Multiple layers feed forward ANN topology was chosen to develop the primary triage prediction model and map our input data to fit the output data which is a nonlinear problem. The practical activation function used by the neuron is the step, sign, linear, and sigmoid functions. The number of neurons in the hidden layer was chosen by trial and error (Asiltürk and Çunkaş 2011; Kisi 2004; Das et al. 2003). The number of the neurons should not be set to a high number to avoid over-training or set to a low number that will cause insufficient generalization. The optimal topology is three layers: one in each of the input, hidden, and output layers. The number of neurons in the hidden layer was set to 12 neurons. The optimal parameter was set randomly to give the best convergence with less error. It is worth to mentioned that inappropriate setting of the initial parameter will lead to different types of problems, like divergence, slow convergence, or local minimum trapping (Kermani et al. 2005).

**Figure 2:**
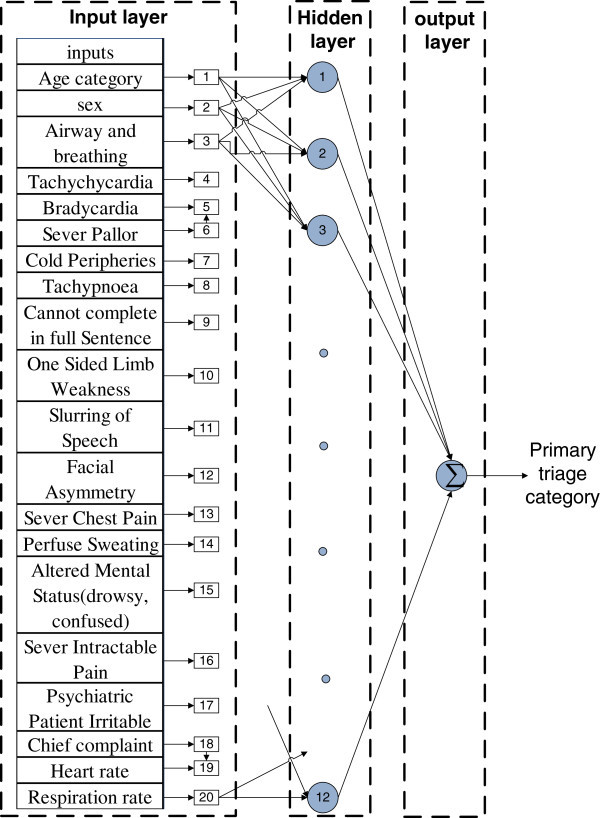
**ANN layers used in the construction to predict the triage category.**

The parameters were set to zero as the training performance goal, a zero sum square error and 0.001 learning rate. Figure 2 shows the three multiple layer networks with twelve neurons in the hidden layer that were chosen to develop the model. At the beginning, the network was created and the weights and biases were initialized. After that, the network was trained according to the inputs and given outputs in order to minimize the network performance capacity and weights. Biases were adjusted by minimizing its mean square error. Figure 2 shows the network used in this work. It has three layers and connects between the input, hidden and the output layer. The inputs to the ANN model consist of 20 variables start with the age category and ending with the respiration rate.

##### Adaptive network-based fuzzy inference systems method

A hybrid intelligent system is one of the best solutions in data modeling, where it’s capable of reasoning and learning in an uncertain and imprecise environment (Bodyanskiy and Dolotov 2010). It is a combination of two or more intelligent technologies. This combination is done usually to overcome single intelligent technologies.

The fuzzy system cannot learn or adapt by itself to the new environment, while the ANN is ambiguous to the user. By combining these two methods, the ANN becomes more transparent and the fuzzy system takes on the ability of learning. With this combination, a more effective model in the medical domain could be built (Pandey and Mishra 2009; Mangalampalli et al. 2006) with quick and accurate decision-making. Though, this combination reduces some disadvantages, still some others, like the IF-THEN rules in a fuzzy set have to collect anything through what is called ‘knowledge acquisition’ from an expert. This knowledge has some variety of different experts, and the acquisition is considered time-consuming. Besides this, the fuzzy set is considered as a deterministic process for its membership parameters.

**Figure 3:**
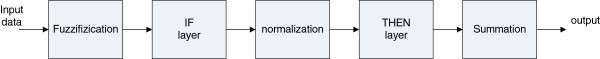
**Main layers in the ANFIS.**

ANFIS is an example of a hybrid intelligent system proposed by Jang (Jang 1992) solved these problems. It depends on data that learn the rules and membership functions. This Inference Systems model for ANFIS uses a first-order Takagi-Sugeno-Kang (TSK) system as inference to generate the if-then rule to build the model that maps input to output, which has high efficiency (Shuangwen and Gao 2006). ANFIS architecture has five layers in its construction (Benmiloud 2011) and its flowchart is shown in Figure 3.

The input data and output data were fed into the ANFIS model to extract the rules. The ‘fuzzification’ layer is set and adapts the parameters for the chosen membership. After that, the strength firing layer is coming and it represents the IF conditions to set the rules. The output of the firing strength is normalized in the normalization layer. Before the final layer, there is another adaptation layer that works as a ‘defuzzification’ layer of the rules, where the surgeon model parameters are tuned to derive the best matching between input and output (Mitra et al. 2007).

The most popular learning algorithms is the hybrid algorithm proposed by Jang (Jang 1991). It is used to adapt the parameters in the adaptive network. This algorithm is a combination of Steepest Descent and Least Squares Estimation (LSE). In this learning algorithm, there are two passes: the forward and feedback passes. LSE is used during the forward pass to tune consequent parameters, and Steepest Descent is used during the backward pass to tune the antecedent parameters. For each input, the experimental membership function was set to the Gaussian type. While the output membership function was set to the linear type.

After training, the parameter of the membership was adapted to give better matching between input and output, which lead to changing the initial shape of the membership. The more changing of the membership shape before and after the training represents the most effective variables in constructing the model. The hybrid algorithm was used to adapt the membership parameter and the Sugeno polynomial parameters. The step-size adaptation parameter was initialized to 0.01. Fuzzy subtractive clustering has been used to find the fuzzy rules for the ANFIS model. The radius of the clustering was changed until 12 rules were obtained, to let it comparable to the number of neurons in the ANN.

Both ANN and ANFIS model were developed using MATLAB (The Mathwork Inc.). The maximum training epoch was 1500. In the post-processing step, the decimal values were removed from the output by rounding the values to the integer number.

##### Evaluation of the triage prediction model

The evaluation of the prediction model was done by using statistical measurements. These measurements are root mean square error (RMSE), percentage root mean square error, and the accuracy (%RMSE). The triage prediction category from both systems was compared to the triage category diagnosed by the medical officer in the medical records. The triage category `gold standard in this study. The RMSE and %RMSE and the accuracy are presented in Eq (1) and Eq (2), respectively.12

where *y’* is the predicted target value, *y* is the actual output value and *n* is the number of data items.

Usually the statistical method gives us the average accuracy or average error. It is useful if we can find which class has confused with which during the test. To find this we need to use the confusion matrix. It shows which class has been classified properly or almost properly and which have confused with another class (Klopotek et al. 2004). To use the confusion matrix we need to have a reference standard to compare with this technique. The reference standard in this work is the triage category made by the medical officers.

From the confusion matrix, specificity and sensitivity are calculated where 100% means that the test recognizes all actual negatives or means no positives are misclassified; a positive result in a high specificity test is used to confirm the disease or the class. It is calculated by dividing the true negative classes over summation of true negative and false positive. At the same time sensitivity of 100% means that the test recognizes all actual positives negatives. Thus, in contrast to a high specificity test, negative results in a high sensitivity test are used to rule out the disease (Gosztolya et al. 2009). It is calculated by dividing the true positive classes over summation of true positive and false negative. The accuracy will be evaluated by measuring specificity and sensitivity for binary classification (Liu and Yuan 2009).

Not only statistical measurements should be used to evaluate the model prediction acceptability, but also the ability of the model to predict the output correctly when the input data is slightly different than the data used in building the model, and has never been seen before (Haykin 2008).For this reason, the even rows of the patients were evaluated as unseen data.

## Results and discussions

### Descriptive data analysis

The triage input data consist of categorical, free text and continuous data. The triage output have three categories namely resuscitation (coded as one), emergent (coded as two) and non-urgent (coded as three). The chief complaints were coded according to the Canadian Emergency Department Information Systems (CEDIS). The general terms of the chief complaint used in the OPTS were added into CEDIS lists with a customized numbers to satisfy the need of model development. These data have numerical values that vary between zero and 854. Mean standard error of mean, standard deviation, range, minimum and maximum of the input and output variables of the primary triage data were calculated and shown in Table 1.

**Table 1:** **Statistical measurement for the input and output variables**

	Age_Cat	Sex	Canadian_code	A_breath	Tachy_C	Brady_C	Pallor	Peripheral	Tachy_P	Dysp	Weak_L	Speech	Facial	chest_p	Sweat	Mental	poly_t	Pain	psy_Irr	thr	trr	Output
Mean	2.62	.56	443.83	.11	.04	.01	.02	.01	.08	.03	.02	.01	.01	.02	.03	.05	.01	.04	.01	80.97	16.59	2.62
Std. Error of Mean	.019	.011	6.662	.007	.004	.002	.003	.002	.006	.004	.003	.002	.002	.003	.004	.005	.002	.004	.002	.706	.143	.015
Std. Dev	.903	.497	314.109	.322	.206	.094	.151	.117	.268	.170	.128	.101	.099	.131	.176	.227	.092	.187	.073	33.297	6.742	.704
Range	5	1	851	2	1	1	1	1	1	1	1	1	1	1	1	1	1	1	1	206	69	2
Min	1	0	3	0	0	0	0	0	0	0	0	0	0	0	0	0	0	0	0	0	0	1
Max	6	1	854	2	1	1	1	1	1	1	1	1	1	1	1	1	1	1	1	206	69	3

### Triage prediction model development using ANN and ANFIS

The prediction model’s performance consist of RMSE, %RME, and the number of the correct classified output that represent the accuracy were tabulated in Table 2. The RMSE, %RME, and the accuracy for the training data were 0.14, 5.7 and 99, respectively, for the ANN model, and 0.85, 32.00 and 96.00 respectively, for the ANFIS model. As for the unseen data, the RMSE, %RME, and the accuracy of the ANN is 0.18, 7.16 and 96.7, respectively; 1.30, 49.84 and 94 respectively, for the ANFIS model.

**Table 2:** **Model performances**

	ANN			ANFIS		
Data set	RMSE	%RMSE	Accuracy	RMSE	%RMSE	Accuracy
Train data set	0.14	5.70	99.00	0.85	32.00	96.00
Unseen test data	0.18	7.16	96.70	1.30	49.84	94.00

**Table 3:** **Confusion matrix for primary triage model using ANN and ANFIS model for training data**

		Actual Classes					
		One		Two		Three	
		ANN	ANFIS	ANN	ANFIS	ANN	ANFIS
Predicted Classes	One	142.00	115.0	6.00	10.0	0.00	0.00
	Two	7.00	8.0	121.00	101.0	0.00	0.00
	Three	0.00	1.0	2.00	9.0	832.0	832.0

The confusion matrix, specificity and sensitivity of the training and unseen data are shown in Tables 3, 4, 5 and 6 as the other indicators of performance of the ANN and ANFIS model. ANN model was more sensitive in triage prediction compared to the ANFIS model for class one and two prediction with the same number of neurons or rules (Tables 4 and 6). However, the sensitivity values for class three prediction were similar for both models. As for the specificity values, ANN and ANFIS models gave similar values around 0.99 for the training data and 0.98 for the unseen data, except for class three ANN model performed better than ANFIS model.

**Table 4:** **Sensitivity and Specificity for primary triage model using ANN model for training data**

	One		Two		Three	
	ANN	ANFIS	ANN	ANFIS	ANN	ANFIS
Sensitivity	0.95	0.93	0.94	0.84	1.00	1.00
Specificity	0.99	0.99	0.99	0.99	0.99	0.96

**Table 5:** **Confusion matrix for primary triage model using ANN and ANFIS model for unseen data**

		Actual Classes					
		One		Two		Three	
		ANN	ANFIS	ANN	ANFIS	ANN	ANFIS
Predicted Classes	One	109.0	63.0	14.0	22.0	0.0	0.0
	Two	22.0	20.0	117.0	66.0	0.0	0.0
	Three	2.0	7.0	7.0	9.0	835.0	835.0

**Table 6:** **Sensitivity and Specificity for primary triage model using ANN model for unseen data**

	One		Two		Three	
	ANN	ANFIS	ANN	ANFIS	ANN	ANFIS
Sensitivity	0.82	0.70	0.85	0.68	1.00	1.00
Specificity	0.99	0.98	0.98	0.98	0.97	0.91

The ANN performed better in all the three statistic measurements for the training and unseen data. ANFIS works as a small window on the model to show which parameters are actually influenced the prediction output. While neural network works as a black box. The parameters of the membership were adapted to give better matching between input and output during ANFIS training. It changed the shape of the membership according to the new adapted parameter. The most effective variable are airway and breathing (variable 1) as its membership shape had significantly changed after training compared to the other variables.

## Conclusions

In this paper we showed the ability of ANFIS and the ANN in modeling the primary triage data. The biomedical text medical data were extracted from the OPTS data sheet from the Emergency Department, UKMMC. The triage decision support system could be a clinically useful tool in emergency medical services. This tool could prompt the emergency department physician to systematically collect pertinent and readily available information, and then provide a patient-specific recommendation based on this information.

The results showed that the RMSE, %RME, and the accuracy for the training data were 0.14, 5.7 and 99, respectively, for the ANN model, and 0.85, 32.00 and 96.00, respectively, for the ANFIS model. As for unseen data, the RMSE, %RME, and the accuracy of the ANN is 0.18, 7.16 and 96.7, respectively; 1.30, 49.84 and 94.00, respectively, for the ANFIS model. The ANN model was more sensitive than the ANFIS model on the training and unseen data set for class one and two prediction. For class three predictions, same values have been achieved for training and unseen data for both models. In term of specificity, ANN model is performing better compared to ANFIS model for training and unseen data.

As for the model accuracy, ANN model works better compared to ANFIS model in triage prediction. We can conclude that ANN model can fit the output better compared to the ANFIS model for the unseen data set. This means that the ANN is better than ANFIS in generalization. It was therefore chosen as the technique to develop the primary triage prediction model. Any future work studies could be focused on the secondary triage data modeling using the OPTS data. This model could be integrated with the secondary triage to give the final triage model to predict the overall triage category to be used as assessment tools for the medical officers in the ED.

## Consent

Written informed consent was obtained from the patient for the publication of this report and any accompanying images.
